# Evidence of human exposure and associated risk factors to Rift Valley fever in selected districts of Central and Western Zambia

**DOI:** 10.1371/journal.pone.0309288

**Published:** 2025-01-24

**Authors:** Chilufya C. Kasongamulilo, Michelo Syakalima, Ngondo Saasa, Henson Kainga, Girja S. Pandey, Andrew N. Mukubesa, Innocent Mwape, Masahiro Kajihara, Ayato Takada, Martin Simuunza

**Affiliations:** 1 Department of Disease Control, School of Veterinary, Medicine, University of Zambia Lusaka, Lusaka, Zambia; 2 Department of Veterinary Epidemiology and Public Health, Faculty of Veterinary Medicine, Lilongwe University of Agriculture and Natural Resources, Lilongwe, Malawi; 3 Centre of Infectious Diseases and Research in Zambia, Lusaka, Zambia; 4 Division of Global Epidemiology, International Institute for Zoonosis Control, Hokkaido University, Sapporo, Japan; 5 International Collaboration Unit, International Institute for Zoonosis Control, Hokkaido University, Sapporo, Japan; 6 One Health Research Centre, Hokkaido University, Sapporo, Japan; 7 Africa Centre of Excellence for Infectious Diseases of Humans and Animals, University of Zambia, Lusaka, Zambia; Clinton Health Access Initiative, UNITED STATES OF AMERICA

## Abstract

Rift Valley fever (RVF) is an important viral zoonotic disease that not only affects ruminants but causes serious morbidity and mortality in humans. In humans, its symptoms range from mild flu-like signs to a severe form such as retinal damage, meningoencephalitis to haemorrhagic fever. In this study, 202 human serum samples were collected from central and western parts of Zambia and tested for RVF-specific antibodies using a commercially available ELISA kit. A pre-tested semi-structured questionnaire was used to collect data for determining hypothesised risk factors for exposure to RVF. The participants enrolled in this study, were only those who are at high risk of RVF infection and were in close contact with animals and animal products. The study revealed an overall seropositivity of 9.90%, occupationally distributed as 16.67% among slaughter house workers, 14.41% among livestock farmers, and 0% among the others (i.e., students, butchery, and farm workers). The prevalence was highest (19.23%) in Sesheke district found in the western part of Zambia, while Chisamba district in central Zambia had the lowest prevalence (1.41). Movement of animals in search of greener pastures was identified as a risk factor to being RVF-seropositive. This suggests that there was silent circulation of the virus in the interepidemic period in the study areas. Therefore, this study recommends that public education of livestock farmers, public health workers, slaughterhouse workers, communities, livestock and veterinary staff needs to be enhanced to increase awareness and preparedness for RVF outbreak in Zambia.

## Introduction

Rift Valley fever (RVF) is an important viral zoonotic disease that affects both humans and animals such as sheep, goats, cattle and camels. It is caused by the Rift Valley fever virus (RVFV), a virus belonging to the order *Bunyavirales*, a member of the *Phenuviridae* family and genus *Phlebovirus*. [1, 2]. It is transmitted mostly via infected mosquito (*Culex* and *Aedes* species) bites and from contact with infected animal tissues and fluids [3, 4]. In humans, symptoms range from mild flu-like to severe forms such as retinal damage, meningoencephalitis and hemorrhagic fever. Case fatality rate among patients with more severe infections can be as high as 14% as was seen in an outbreak in Saudi Arabia in 2000/1 [5].

RVF has become one of the most important zoonoses in sub-Saharan Africa over the last century, causing devastating health and economic impacts on domestic ruminant industries and humans [6]. Socioeconomic impact of RVF was seen in 2006–2007, when Tanzania experienced disruptions in the livestock market value chains, and human and livestock deaths [7]. Apart from that, high levels of malnutrition were recorded due to the inability of people to consume proteins [8]. Individual and national monetary losses during this period were experienced due to reduction in prices during the RVF outbreak, where average losses on bulls and oxen were estimated to be 43 United States Dollars (USD) per animal and an estimated 1740 USD loss on the bulls, cows, heifers, goats and sheep that died during the outbreak [8]. Outbreaks of this disease in cattle were reported in Zambia (1986/ 1987) and Zimbabwe(1950/1951) resulting in numerous losses in livestock [9–11]. In Zambia, sera that was collected in 1978 from 15 different farms showed that cattle and sheep were seropositive for RVFV, indicating a RVF epizootic in Chisamba, Ndola, Lusaka and Mazabuka districts [10]. In 1985 and 1986, a seroprevalence of 22% in animals of Namwala district and Lutale area of Mumbwa district was observed [10]. A prevalence of 9.4% of RVF antibodies was recorded in Lusaka abattoir workers dealing with pigs, while in Mazabuka district 11.38% tested seropositive to RVFV antibodies and of these, only seven had previous contact with cattle. Therefore, transmission was assumed to be by mosquito bites [12].

A compiled meta-analysis report of 17 studies (from countries generally from the horn of Africa, Kenya, Tanzania, Somalia, Yemen, Saudi Arabia and Sudan) showed high economic impacts ranging from $5 to USD 470 million worth of losses due to RVFV infection [13]. In 2016, Niger reported 348 human cases of RVF, and of these, 9.48% resulted in deaths due to the disease [14]. In 2006–2007, Kenya reported 700 human cases with 158 deaths, while during the same period, Tanzania reported 264 RVF human cases with 109 deaths, whereas in 2009–2011 South Africa and Namibia reported more than 250 RVF human cases with 26 deaths [14].

Previous studies have explained the high possibility that RVF is misdiagnosed for other febrile conditions having similar clinical features such as fever, diarrhea and vomiting [15, 16]. Zambia has not recorded any outbreak of RVF in the recent past, and as such little is known about the current seroprevalence and risk factors associated with RVFV infection in human populations in the country, hence the need to carry out this study.

## Materials and methods

### Ethics statement

Participants (18 years and above) signed a written consent to participate in the research and all the information concerning the research was availed to them. Only respondents above the age of 18 were interviewed and sampled. Those below the age of 18 years were considered minors and no ethical clearance was obtained to sample them as a result they were excluded from this study. The study’s ethics approval was obtained from the University of Zambia Biomedical Research Ethics Committee (UNZA-BREC) REF.NO.1294-2020. Approval to carry out research was also obtained from National Health Research Authority (NHRA) and Ministry of Health (MOH).

### Study area

The study was carried out among those in close contact with livestock such as livestock keepers and workers at *slaughterhouses or* abattoirs in selected districts of Western and Central provinces of Zambia. The districts were purposively selected based on previous studies having reported livestock exposed to RVFV [10, 17]. The districts selected were Chisamba (Central province), Mulobezi, Mwandi and Sesheke (Western province). The selection of households, abattoirs and individual participants was done using random selection method.

Chisamba district is located in Central province of Zambia (lattude13⁰, 15⁰ south and longitude of 27⁰, 29⁰ East) with a human population of 160,828 [18]. Chisamba receives an average monthly rainfall ranging from 160–200 mm from November, December, January, February to March (NDJFM) [19]. It is well known for agricultural activities with over 32,000 small scale farmers and 256 commercial farmers [20].

Mwandi and Sesheke districts are adjacent to each other in Western province of Zambia. These two districts have the lowest agricultural potential with less than 800 mm of rainfall per annum and a medium to high risk of drought [21]. The people in the area rely on livestock for income and livelihood. The flood plains and river banks offer a conducive grazing area for the cattle. Apart from cattle, chickens, goats, pigs and donkeys are kept as livestock [22]. Sesheke district has a human population of 72,655 and receives between 100–140 mm of average monthly rainfall in NDJFM [19, 23]. On the other hand, Mwandi district is located on latitude 16⁰ south and longitude 24.6⁰ East. Mwandi has more cattle population of 35,000 compared to the human population of 23,201 showing that livestock keeping is the main occupation of the people living in the area [22]. Currently, the human population of Mwandi stands at 40,418 people. In NDJFM, Mwandi receives 100–120 mm of average monthly rainfall.

Mulobezi district is also located in Western province of Zambia with latitude 16.8⁰ South, longitude 25.2⁰ East. It has a human population of 45,326 and receives 120–140 mm monthly average rainfall in NDJFM [23]. Mulobezi district shares boundaries with Sesheke, Lumpa, Kazungula, Senanga and Mwandi districts. It is called the Land of timber and cattle, because it is known for timber production and cattle rearing. The other main sources of livelihoods for the people in the district are food production and fibre crops cultivation [24].

### Study design and sample size estimation

This was a cross-sectional study targeting individuals in close contact with livestock, including livestock farmers and those who work in slaughter houses and meat processing plants. The sample size was calculated using the following formula for estimating proportions according to Dohoo *et al*. [25] with assumption of a 95% confidence interval, a prior prevalence of 9.4% [12] and a maximum allowable error of 5%, which gave a minimum sample size of 124. This was proportionally allocated to each district based on the total population for each district as follows: Chisamba n = 63, Sesheke n = 27, Mulobezi n = 19 and Mwandi n = 15.

### Sampling and serum preparation

Sample collection was carried out from the 1^st^ of March to the 30^th^ of April 2022. Participants who gave written consent to the study, were asked to respond to the questionnaire and to allow the collection of blood (5 ml) through vein-puncture of cephalic vein by a qualified biomedical technologist in plain vacutainer tubes. Each filled vacutainer tube was assigned a unique identification code according to the districts where they were sampled. Samples were stored on ice in a cooler box until taken to the laboratory, at the University of Zambia, School of Veterinary Medicine. In the laboratory, each sample tube was centrifuged at 2,500 rpm for 15 minutes and then the serum was aliquoted into appropriately labelled cryotubes and stored at -80 degrees Celsius until sample analysis.

A semi-structured questionnaire was administered face-to-face to each sampled person to capture information on potential risk factors for exposure to RVFV. The interviews focused on collection of information on the socio-demographics of the respondents, age and sex, exposure to mosquitoes, any symptoms related to RVF in the past month, attitude, practices and frequency of contact between domestic animals and the presence of wildlife species, work exposure and use of personal protective equipment when handling the animals or meat.

### Laboratory analysis of the samples

#### Competition multi-species ELISA

Laboratory analysis was carried out at the Public Health Laboratory in the Department of Disease Control, School of Veterinary Medicine, University of Zambia in Lusaka. The ID Screen RVF Competitive Multi-Species ELISA kit (Innovative Diagnostics, Grabels, France) that detects serum/plasma antibodies (mainly IgG and IgM) against the RVFV nucleoprotein (NP) was used for screening the samples [26, 27]. This assay has sensitivity and specificity of 91%– 100% and 100% (95% CI: 99.58%– 100%), respectively, based on livestock samples collected (bovine, ovine, caprine) [28]. Therefore, it can detect antibodies against RVFV regardless of the species tested and it has been previously used to detect of antibodies against RVFV in humans [26, 29, 30]. This ELISA method has been proven to be a low-cost surveillance tool for the African context with better accuracy than other ELISA methods, with a specificity similar to that which was reported in 2005 of 0.997, but with a slightly lower sensitivity of 0.96 that was reported [31]. The ELISA can either contain 384 tests (4 plates) or 960 tests (10 plates) per kit for a minimum price of approximately 2100 USD [31]. The ELISA was carried out according to the manufacturer’s instructions. Plates were read at an optical density (OD) of 450 nm using a microplate immunoskan reader (Biological Diagnostic Supplies Limited, Scotland, United Kingdom).

#### Data analysis

The data generated was stored and cleaned in Microsoft Excel 2016^®^ before being transferred to SPSS statistical package version 21(IBM, USA) for descriptive and inferential statistical analysis. Percentages and frequencies of each independent variable were calculated. Bivariate analysis of association between possible risk factors and occurrence of RVFV antibodies (dependent variable) was carried out using Fisher’s Exact test., where applicable. A stepwise binary logistic regression model was used to find determinants (predictors) of people being seropositive to RVF. All variables with p-values less than or equal to 0.250 in the bivariate analysis were included in the model. The logit link function returned the coefficient, p-value, odds ratio (OR) and 95% lower and upper confidence limit values for the OR. Criteria used in determining whether the constructed model adequately fitted the data were, a non-significant Hosmer and Lemeshow Test (p > 0.05) and a significant Omnibus Test of Model Coefficients (p < 0.05). All statistical tests were considered significant at p ≤ 0.05.

## Results

### Seroprevalence for RVF

Serological data based on the RVF Competition Multi-Species ELISA are shown in Table 1. In this study, 20 out of 202 participants were seropositive, giving an overall seroprevalence of 9.9% Males had a significantly higher seroprevalence of RVF than females (p = 0.014). There was also a significant difference in seroprevalence of RVF with age, with the prevalence being higher among those who were older than 56 years of age. The seroprevalence was significantly higher in Sesheke (p = 0.003) than in other districts sampled. Only livestock farmers and slaughterhouse workers were found positive, with the seroprevalence being significantly higher among the former than the latter (p = 0.025). There was no significant difference in seroprevalence among those who reported being exposed to mosquito bites and those not exposed (p = 0.646), nor was there any significant difference in prevalence among those that used mosquito nets or repellents (p = 0.893) as shown in Table 1.

**Table 1:** Prevalence of RVF according to demographic, study site and occupational characteristics of respondents.

Variable	Categories	*n*	Number of positives IgG	Prevalence %	p-value
Gender	Male	159	20	12.6	**0.014**
	Female	43	0	0.0	
Age	≤25 years	69	0	0.0	**0.009**
	26–35	46	5	10.9	
	36–45	40	6	15	
	46–55	28	5	17.9	
	≥56 years	19	4	21.1	
Study site	Sesheke	78	15	19.2	**0.003**
	Chisamba	71	1	1.4	
	Mulobezi	28	2	7.1	
	Mwandi	25	2	8	
Occupation	Livestock farmer	111	16	14.4	**0.025**
	Student	31	0	0.0	
	Slaughter man	24	4	16.7	
	Butchery	20	0	0.0	
	Farm worker	17	0	0.0	
Mosquito exposure	Yes	164	17	10.4	0.646
	No	38	3	7.9	
Use of Mosquito net/Repellent	Yes	125	12	9.6	0.893
	No	78	8	10.3	

Study participants reported having experienced a number of symptoms during the month before the study (Table 2). However, as shown in Table 2, none of these symptoms was significantly associated with being seropositive to RVF antibodies, except for dizziness, where the prevalence was significantly higher among those that reported feeling dizzy in the past one month than those that did not (p = 0.038).

**Table 2:** Sero-prevalence of RVF according to symptoms reported by the respondent in the past month.

Variable	Categories	*n*	Number of positives IgG	Prevalence (95% C.I)	p-value
Flu-like fever	Yes	103	11	10.7	0.705
	No	99	9	9.1	
Headache	Yes	118	10	8.5	0.279
	No	84	10	11.9	
Neck stiffness	Yes	21	3	14.3	0.477
	No	181	17	9.4	
Vomiting	Yes	24	3	12.5	0.650
	No	178	17	9.6	
Loss of appetite	Yes	61	6	9.8	0.984
	No	141	14	9.9	
General aches and pains	Yes	77	9	11.7	0.116
	No	123	11	8.9	
Dizziness	Yes	52	9	17.3	**0.038**
	No	150	11	7.3	
Blurred or decreased vision	Yes	24	2	8.3	0.784
	No	178	18	10.1	

The results of the association between being RVF-seropositive and types of livestock kept, livestock management and behavioral risk factors are shown in Table 3. From the results obtained, those who reported that their cattle were moved in search of pasture and water and grazed with wildlife were statistically significantly associated with the RVF antibody prevalence. Furthermore, the participants who reported drinking raw milk from cattle were also associated with being RVF-seropositive. All the other factors shown in the table were not significantly associated with participants’ RVF seropositivity.

**Table 3:** Sero-prevalence of RVFV according to the type of livestock, management and behavioral risk factors of respondents.

Variable	Categories	*n*	Number of positives IgG	Prevalence (95% C.I)	p-value
Live near dambo/swamps	Yes	116	15	12.9	0.094
	No	86	5	5.8	
Keep any livestock	Yes	171	17	9.9	0.964
	No	31	3	9.7	
Type of livestock kept	Cattle & goats	60	10	16.7	0.092
	Cattle	56	6	10.7	
	Cattle, Goats & Pigs	31	0	0.0	
	Cattle, Goats & Sheep	18	1	5.7	
	Goats	9	0	0	
Animals move for pasture and water	Yes	107	16	15	**0.004**
	No	67	1	1.5	
Graze with wild animals	Yes	132	17	12.9	**0.014**
	No	44	0	0.0	
Interaction with the wild animals <30days	Yes	142	14	9.9	0.893
	No	33	3	9.1	
Raw milk intake	Yes	132	19	14.4	**0.003**
	No	70	1	1.4	
Milk from	Cow	124	18	14.5	0.875
	Cow & Goat	8	1	12.5	
Preparation of raw meat	Yes	120	13	10.8	0.591
	No	82	7	8.5	
PPE	Yes	55	2	3.6	0.251
	No	105	12	11.4	

In this study, 107 participants let their animals move for pasture and water and 14.9% of these were seropositive. Of the 67 participants that did not allow their animals to move in search for pasture and water only, 1.5% were positive. There was a statistical difference in prevalence between those that allowed their animals to go in search of pasture and those that confined their livestock (p = 0.040). The livestock farmers that let their livestock to go out and graze with wild animals had a significantly higher seroprevalence than participants that did not allow their livestock to interact with wild animals. Of the 132 that consumed raw milk, 19 were seropositive for RVF while only one of the 70 participants that did not consume raw milk was seropositive. Thus there was a statistical difference between those that consumed raw milk and those that did not (p = 0.003), whereas no significant difference was found between the participants that consumed raw milk from cows (14.5%) and those that consumed from both cow and goat (12.5%). All the other management and behavioural risk factors showed no statistical significance to RVF seroprevalence.

### Maximum likelihood estimates of people being seropositive to RVF

A forward stepwise binary logistic regression model was used to determine predictors of people being seropositive for RVF on ELISA. A non-significant Hosmer-Lemeshow goodness-of-fit statistic (p = 0.902) and a significant Omnibus Test of Model Coefficients (p < 0.001) were obtained, indicating that the model fitted the data. It was found that movement of animals from one pasture to another in search of food and water was a significant predictor of people being positive RVF seroprevalence (Table 4). All other variables were not significant predictors of people being seropositive.

**Table 4:** Maximum likelihood estimates of predictors of an individual being seropositive to RVFV.

Variable	Categories	Odds Ratio	95% C.I.for Odds Ratio		p-value
			Lower	Upper	
Animals move for pasture and water	Yes	11.6	1.5	89.7	0.019
	No				
Constant		0.02			< 0.001

## Discussion

The aim of this study was to determine whether there was evidence of exposure to RVFV among people who were in contact with livestock and livestock products in the selected districts of Western and Central provinces of Zambia and to identify potential risk factors associated with this exposure. This is because RVF is an important re-emerging zoonotic diseases with recurrent but irregular intervals of occurrence [32]. This study revealed that people living in the study areas had been exposed to RVFV, with Sesheke having the highest, while Chisamba had the lowest prevalence. In 2018, antibodies to RVFV were detected in cattle in Sesheke suggesting a circulation of the virus in that area [17]. In 1974, Zambia reported its first-ever RVF outbreak in Chisamba district [10] and the present study shows that the virus is indeed still circulating in the district. Mulobezi district recorded two seropositive cases of RVF in this study. The district is located adjacent to both Mwandi and Sesheke districts and transmission of the virus into Mulobezi district is expected, especially that it was previously part of Sesheke district. Previous studies done in other provinces (Southern Provence (Mazabuka district) and Lusaka province (Lusaka district)) also indicated a presence of antibodies to RVFV [12]. Taken together, these data suggest that RVF may be prevalent in a number of districts in the country [33, 34].

In this study, the bivariate analyses of a number of variables were found to be significantly associated with people being seropositive to RVFV. Such variables included gender, age, study site, occupation, movement of animals for pasture and water, animals grazing together with wild animals, drinking of raw milk. and, dizziness. This study included a higher proportion of males compared to females. This might have increased the chances of males being seropositive for RVF since the number of study samples was relatively low. However, previous studies, have shown that being male was a significant risk factor for RVFV infection [35]. This might be because males were more involved in the livestock handling, heading of cattle and slaughtering of animals and thus were more in contact with the animals than the females. A study done in Kenya also reported that males were three times more likely to have antibodies to RVFV than the opposite sex [36]. However, a study on social culture in Africa showed that women were most likely to be more exposed to RVFV due to frequently spending more time in animal care [37]. In some parts of Sub-Saharan Africa women work more with animals and animal products like milking and slaughtering of small animals such as goats and sheep [37]. Gender roles therefore likely cause differential exposure to RVFV depending on areas and cultural backgrounds.

This study indicated that interaction between livestock and wildlife might be associated with seropositivity, suggesting that there is a potential spill over of RVFV into livestock, then humans, especially during outbreaks. However, more studies are needed to estimate the force of infection in livestock, wildlife and humans and to understand RVFV transmission dynamics [38]. In this study, wild animals that interacted with the participant’s livestock included impala, kudu, buffalo, Duiker, monkeys, tigers, elephants, monkeys, hyenas, lions, zebra, gazelle, fox, and hare. In other countries, wildlife such as African buffaloes have been suggested to help and play a role in virus amplification during the interepizootic period of RVF [38, 39]. The ingestion of raw milk also has potentially large consequences on public health and this is a common practice among rural people in Zambia. Indeed, it is significantly associated with RVFV seroprevalence in the present study. Other studies have also suggested that RVFV could also be contracted through the ingestion of raw milk from infected animals [40, 41]. A study done in Tanzania, showed presence of RVF nucleic acids in milk of which 21% of the 14 cattle milk samples collected were positive [42].

A Multi-variate analysis in this study showed that movement of animals from pasture to pasture in search of food and water was the only factor that was a predictor of potential RVFV infection. Therefore, it can be concluded that those people who were in close contact with animals that were left out in search of pasture and water were more likely to be seropositive for RVF than those who did not. Previous studies have shown that human RVFV infection was associated with the proximity of infected animals to humans and animal movements [43]. Uncontrolled movement of infected animals from enzootic areas has been shown to trigger outbreaks in other countries [44–46]. Those that were in close contact with animals that were let to move about for pasture and water in this study were 11.6 times more likely to be seropositive to RVFV than those that did not. A previous study done in Tanzania showed that movement of animals played a key role in the spread of the infection [47]. Disease free areas are therefore under threat due to increased national and transboundary nomadic and commercial animal movements [48]. All the other variables that were significantly associated with RVFV seropositivity in the bivariate analysis were not significant predictors in the multivariate analysis. This could be attributed to low sample size and or confounding factors.

In conclusion, the estimated RVF seroprevalence in this study was not that much different from that recorded in a previous study (9.2%) [12]. This study has shown that RVFV had been circulating in parts of central and western provinces of Zambia. Further studies are therefore needed to identify when transmission occurred by determining the time of the day people were exposed to mosquito bites. This would allow narrowing down virus transmission to the biting habits of the main vector *Aedes aegypti*. A similar and more comprehensive study with a larger sample size should be extended to other provinces to ascertain the distribution and seroprevalence of RVF across the country. The information gathered in this study will assist in decision-making for developing strategies and resource allocation for surveillance of RVF and also for potential zoonotic disease control interventions.

## Supporting information

S1 Appendix Questionnaire: Enquiry on possible exposure to Rift Valley fever in selected districts in Zambia. (DOCX)

S2 Appendix Coded data: Enquiry on possible exposure to Rift Valley fever in selected districts in Zambia. (XLSX)
