# Supplementary material for: Evidence of human exposure and associated risk factors to Rift Valley fever in selected districts of Central and Western Zambia
Source: PLoS One. 2025 Jan 24;20(1):e0309288. doi: 10.1371/journal.pone.0309288 (PMC11760628; doi:10.1371/journal.pone.0309288)
Supplement: S1 Appendix — (DOCX) [file pone.0309288.s001.docx]

**QUESTIONNAIRE**

Enquiry on possible exposure to Rift Valley Fever in selected districts in Zambia.

Study Title: **SEROPREVALENCE OF RIFT VALLEY IN HUMANS AND THE ASSOCIATED RISK FACTORS IN SELECTED DISTRICTS OF CENTRAL AND WESTERN ZAMBIA**

**Aim of the study**: The aim of this study was to determine the extent of seroprevalence to RVFV in humans and to identify the associated risk factors associated with this exposure to the virus in selected districts of Central and Western Zambia

**Specific objectives**

1. To determine the seroprevalence of RVFV in humans in the selected districts of Central and Western Zambia
2. To determine risk factors associated with seropositivity to RVFV in humans in the study areas.

*This questionnaire should be completed by all individuals work in abattoirs and /or are livestock farmers*

| Interviewer’s name _________________________    Date and time of interview ______________ at _____________  *date time*  Interview number /___/___/  Village/farm: _____________  Geographical coordinates: Latitude: _____________  Longitude: _____________    Person interviewed: self ⬜ other ⬜ (please specify) __________________ |
| --- |

# Section 1 – Personal details

1. Sex M  F 
2. Age ______ years
3. Occupation *(describe what person actually does)*

____________________________________________________

____________________________________________________

# Section 2 - Clinical details

1. For the past six months have you been exposed to mosquitos

Yes -1- No -2-

1. Do you use any mosquito repellant or mosquito net?

Yes -1- No -2-

| 1. . Have you had any of the following symptoms in the past one month? | |  |  |  |  |  |
| --- | --- | --- | --- | --- | --- | --- |
| ***(if symptoms still continuing code 9999)*** |  |  |  |  |  |  |
| Yes | No |  | DK | Duration |  |  |
| Flu-like fever 1 | 2 |  | 9 | _____________ |  |  |
| Headache 1 | 2 |  | 9 | _____________ |  |  |
| Neck stiffness 1 | 2 |  | 9 | ____________ |  |  |
| Vomiting 1 | 2 |  | 9 | _____________ |  |  |
| Loss of appetite 1 | 2 |  | 9 | _____________ |  |  |
|  |  |  |  |  |  |  |
| General aches and pains 1  Dizziness 1  Blurred or decreased vision 1 | 2  2  2 |  | 9  9 9 | ­­­­­­­­­­­­­­­­­______________  ______________ |  |  |
|  |  |  |  | _______________ |  |  |
| Other symptoms (please describe) 1 | 2 |  | 9 | ________________ |  |  |

1. Do you stay near swamps/ damboos? Yes -1- No -2-
2. Do you keep any ruminant livestock? Yes -1- No -2-

If ‘Yes’, how many? Cattle: _____ Sheep: _____ Goats: _____

1. Do you move the animals from one area to another during the year in search of pasture and/water? Yes -1- No -2-
2. Does your livestock share same grazing lands with wild animals? Yes -1- No -2-
3. Have you been in contact with any of these animals in the past 30 days?

Yes -1- No -2- (**go to Q13)**

1. What animals are those______________________________________________

_________________________________________________________________

1. Have you ingested any raw milk from the animals in the past 30 days?

Yes -1- No -2- (**go to Q15)**

1. From which animal/s was that milk from? ______________________________
2. Have you been involved in preparation of raw meat in the past 30 days? Yes -1- No -2- (end of interview)
3. Do you use any type of personal protective clothing when handling the meat? Yes -1- No -2- (end of interview)
4. What type of PPE do you use? ___________________________________

**This completes the interview. Thank you very much for your cooperation.**
